# Editorial: Innovative technologies and materials for treatments of hepatobiliary and pancreatic diseases

**DOI:** 10.3389/fbioe.2023.1260144

**Published:** 2023-08-03

**Authors:** Di Li, Chao Zhao, Weiguo Xu, Yahui Liu

**Affiliations:** ^1^ Department of Hepatobiliary and Pancreatic Surgery, General Surgery Center, The First Hospital of Jilin University, Changchun, China; ^2^ Changchun Institute of Applied Chemistry, Chinese Academy of Sciences, Changchun, China; ^3^ Department of Chemical and Biological Engineering, The University of Alabama, Tuscaloosa, AL, United States; ^4^ Center for Convergent Biosciences and Medicine, University of Alabama, Tuscaloosa, AL, United States; ^5^ Alabama Life Research Institute, University of Alabama, Tuscaloosa, AL, United States

**Keywords:** hepatobiliary and pancreatic diseases, surgery, chemotherapy, radiotherapy, immunotherapy, advanced biomaterial

## 1 Introduction

Treatment for hepatobiliary and pancreatic diseases can be categorized into surgical and non-surgical therapy. With the continuous innovation and development of surgical techniques, hepatobiliary and pancreatic diseases can now be effectively treated with a high cure rate. Unfortunately, the rapid progression of hepatobiliary and pancreatic cancers often leads to many patients being diagnosed at advanced stages, resulting in the loss of opportunity for surgical intervention. Non-surgical treatment methods, including chemotherapy, radiotherapy, and immunotherapy, have emerged as primary treatment options. However, these treatments come with severe side effects that limit the patient’s prognosis. This Research Topic focuses on the development of advanced surgical techniques and the application of new advanced biomaterials for non-surgical therapies of pancreatic diseases, and four articles are presented.

## 2 Advanced surgical techniques

Over the decades, minimally invasive surgery on pancreas resection has continuously evolved as a routine procedure, such as laparoscopic and endoscopic surgery, to reduce blood loss, alleviate pain, and improve visualization, thus significantly reducing the risk of surgery. However, the pancreatic leak rate has remained constant, varying between 20% and 40% or sometimes higher. Sheen et al. investigated the effects of using an innovative endovascular stapler, AEON™, on pancreatic leak rates and other outcome measures. An analysis of patients undergoing distal or lateral pancreatectomy using the AEON™ Stapler to transect the pancreas showed a significant reduction in the incidence of pancreatic fistula, length of hospital stay, and tended to reduce the composite complication index.

The incidence of acute pancreatitis (AP) continues to rise, with walled-off pancreatic necrosis (WOPN) being its severe complication and its potential to lead to the destruction of main pancreatic duct (MPD). Meng et al. retrospectively studied the efficacy and safety of their team’s endoscopic passive transpapillary drainage (PTD) to pancreatic duct disruption. All patients with symptomatic WOPN and complete MPD disruption underwent endoscopic PTD with FCSEMS and plastic pancreatic stent placement. The clinical symptoms connected with WOPN disappeared postoperatively in all three patients. During the 4–18 months follow-up, all patients recovered uneventfully without collection recurrence or other complications, such as gastrointestinal bleeding or reinfection.

## 3 Advanced biomaterials for non-surgical therapies

Drugs are essential for non-surgical therapies for inflammatory conditions and even tumors. Nanomedicine carriers composed of advanced biomaterials have created satisfactory achievements, ranging from improved efficacy, physicochemical properties, and pharmacokinetics to safety. Cai et al. summarized the targeting and functional effects of biomaterials-based nano-agents specifically for AP therapy. Regarding targeting effects, the active and passive targeting of biomaterials-based nanocarriers was categorized. Functional biomaterials based on poly(lactic-*co*-glycolic acid) and liposomes form a significant part of the passive targeted treatment of AP. The biomaterials-based nanocarriers exhibited active targeting properties by specific ligands, such as specific peptides to recognize and bind with corresponding receptors expressed in specific cells in the inflammatory sites, resulting in enhanced drug accumulation. Biomaterials-based nano-agents, which mimic enzyme activities or have different chemical groups on the surface, have been classified into other functional effects in AP therapy. Biomaterials-based nanocarriers exhibited multiple advantages in precise AP therapy.

Biomaterials-based nanocarriers also play a crucial role in chemistry therapy. Li et al. developed an arginine-glycine-aspartic (RGD) peptide-modified nanogel (RGD−polyethylene glycol−poly(L-phenylalanine-*co*-L-cystine)) to deliver vincristine for antitumor therapy. RGD peptide on the surface of this nanoplatform binds to integrin a_ν_β_3_, which overexpressed on the tumor cells, thus improving targeting and antitumor efficacy.

## 4 Conclusion

In summary, this Research Topic contains advanced surgical techniques and the application of new advanced biomaterials for non-surgical therapies for pancreatic diseases. Advanced minimally invasive surgery and biomaterials-based nanomedicine therapy are rapidly evolving, reducing patients’ pain and improving their quality of life.

